# Edpuzzle versus Moodle: Learning Tools in Pediatric Dentistry Practice: A Study Pilot

**DOI:** 10.3390/healthcare10122548

**Published:** 2022-12-15

**Authors:** Nuria Esther Gallardo-López, M. Esperanza Sánchez-Sánchez, Gonzalo Feijóo-Garcia, Antonia M. Caleya

**Affiliations:** Department of Dental Clinical Specialties, Faculty of Dentistry, Complutense University of Madrid, 28040 Madrid, Spain

**Keywords:** Flipped Learning, Pediatric Dentistry, Edpuzzle, Moodle

## Abstract

The aim of the present study was to compare the results of two educational platforms for the development of Flipped Learning (FL) in the preclinical practices of paediatric dentistry: Edpuzzle and Moodle 3.4. Methods: Fifty students filled out a questionnaire on knowledge of pulp treatments in primary dentition (Pre-Q). They were divided into two groups: one watched a video on the pulpotomy technique before preclinical practice using Moodle 3.4 (group A) and the other used Edpuzzle (group B). On the day of practice, the students resolved any doubts with the teacher. Next, they performed a pulpotomy on an artificial tooth and answered the questionnaire again (Post-Q) together with a satisfaction survey. Results: In both groups, an increase in the number of correct answers was found in the Post-Q compared to the Pre-Q (*p* < 0.001), but with no significant differences between groups. The pulpotomy practice ratings were higher in group B, which used Edpuzzle (*p* < 0.001). In the satisfaction survey, we only found significant differences (*p* = 0.003) in access to the video, since 100% of the students in group A found it easy to view it through Moodle, unlike in group B. Conclusions: Our results suggest that the use of educational platforms specially designed for FL, such as Edpuzzle, can improve the qualifications of students in paediatric dentistry practices.

## 1. Introduction

The flipped classroom or “Flipped Learning” (FL) is a pedagogical model based on reversing the traditional structure of the expository face-to-face class through the use of information and communication technologies [1]. This involves a change of roles between teacher and student. The acquisition of new knowledge begins before the face-to-face class, for which the teacher provides the students with the necessary tools, which generally require the support of educational platforms. Initially, for the teacher, starting to use FL can be more complex, since it requires elaboration of all the material that must be provided to the student. However, once all the material has been prepared, it can be more versatile and make it easier to evaluate the student. Therefore, for the development of FL, it is necessary to previously establish the learning objectives, plan the activities to be carried out, and create materials for prior delivery to the student. The content can be accessed through the Internet.

Multiple educational platforms have been created for the development of FL, such as Edpuzzle, Panopto, eduCanon, Google Forms, and Socrative. After analysing them, we selected Edpuzzle to carry out our study due to its accessibility, ease of use, and the means by which it allowed the evaluation and analysis of the classes.

Edpuzzle is a free application that stands out for its versatility in creating audio-visual content, which students can view from any digital device [2]. The most important advantage of Edpuzzle is that it allows the personalisation of videos already prepared by others. Once the video has been selected, which can come from repositories such as YouTube, or own production, it can be edited to select the part of interest [3]. In addition, teachers can record their voice over the video to add an introduction, explain the content or add subtitles or comments. By pausing the video, the student can be asked to answer some questions, preventing the video from advancing if they are not answered.

There are two types of accounts on Edpuzzle: one for teachers and one for students. The content of the class is private and can only be viewed by the teacher and the students who are part of it. Students can watch the videos as many times as they want, pause them, rewind them, as well as answer the questions. These questions can serve as feedback for the teachers since they know how many times the students had to watch the different parts of the video to do the tasks and, thus, they know which parts were more difficult to understand. In addition to questions, the teacher can add additional searches to obtain answers and make comments. These activities can be carried out during the face-to-face class or before it, and so would be applying FL.

The present study was carried out in the Dentistry Degree of the Complutense University of Madrid (UCM), which uses the Moodle 3.4 telematics teaching platform called Virtual Campus (VC), which is used in a private network regime.

Moodle (Modular Object-Oriented Dynamic Learning Environment) is a very useful software tool for teaching. Its name indicates that “learning objects” are created by which the teacher guides the students, thus enabling their self-learning. Moodle has spread exponentially in schools around the world. It allows for the management of subjects as well as other utilities, from uploading multimedia content (e.g., videos, images, and notes), evaluating different student tasks, to taking online exams. It is an ideal tool to manage the organisation of educational communities and allow the different members to work and communicate. As we pointed out previously, this tool allows us to post contents of our subject before the traditional expository class, so it is a useful tool to apply FL [4].

During the 2018–2019 academic year, an educational innovation project was carried out on the incorporation of FL in the preclinical practices of Paediatric Dentistry II in the Dentistry Degree at the UCM. The results indicated that the visualisation of videos through the CV (Moodle) before the preclinical practices improved the acquisition of theoretical knowledge and was very satisfactory for the students [5]. However, in the satisfaction survey, 85% of the students answered that trying other platforms more suitable to FL for viewing the videos would be very useful and interesting.

Our working hypothesis was based on the fact that the effectiveness of the FL teaching methodology is influenced by the educational platform used.

The main objective of our study was to compare Edpuzzle and Moodle used as platforms to apply FL. The secondary objectives were to determine: (1) if this learning improved when introducing FL compared to the group without FL; (2) if technical skills improved by applying FL with the two indicated platforms or without FL; and (3) student satisfaction with the methodology used.

## 2. Materials and Methods

This study was approved by the Vice President for Quality of the UCM with reference number 258 for the 2019–2020 call for educational innovation projects at the UCM. It was carried out by professors of Paediatric Dentistry II in the 4th year of the Degree in Dentistry at the UCM. It is an analytical study, and we compared two computer tools to determine how they influenced learning.

The students were informed of the development of this study and participated voluntarily, with the right to withdraw at any time without any consequences. It was explained to the students that they would have to watch a video through a computer application. In the first session of the course, the selection of the sample was made through a questionnaire to rule out students who had previously studied the subject or had prior knowledge about pulp treatments in temporary dentition. In the degree of Dentistry at the UCM, pulp treatment is studied in the subject Pediatric Dentistry II in the fourth year.

The sample consisted of 50 students and was randomly divided using the Random Number Generator Pro software, version 213 (Segobit Software Inc. Redmond, Washington, USA) into two groups of 25 students, assigning a number to each student. Afterwards, the 50 students completed a knowledge questionnaire created by the authors (Pre-Q) with 10 specific questions about the pulpotomy technique. Each correct question was weighted with 1 point, with the maximum score being 10 (Table 1).

In order to apply the FL teaching methodology, the teachers created a video with theoretical and practical content on the pulpotomy technique in primary dentition that answered the Pre-Q questions and contained demonstrative images, step by step, of the technique. Students were explained the obligation to watch the video before the next practice. They were also informed of the distribution in groups (A and B) and the methodology of access to the video. The students of group A viewed it through Moodle 3.8, on the page of the virtualised subject in the VC—UCM. However, the students in group B received by email the link to a virtual class in Edpuzzle that contained the same video. Using the tools offered by this application, pauses with comments and multi-choice questions about its content were incorporated throughout. Timing was synchronised so that all students had the same access time to the video on their respective platforms (for one week before the preclinical practice). The number of views was not limited. Students were asked not to share the video with peers.

**Figure 1:**
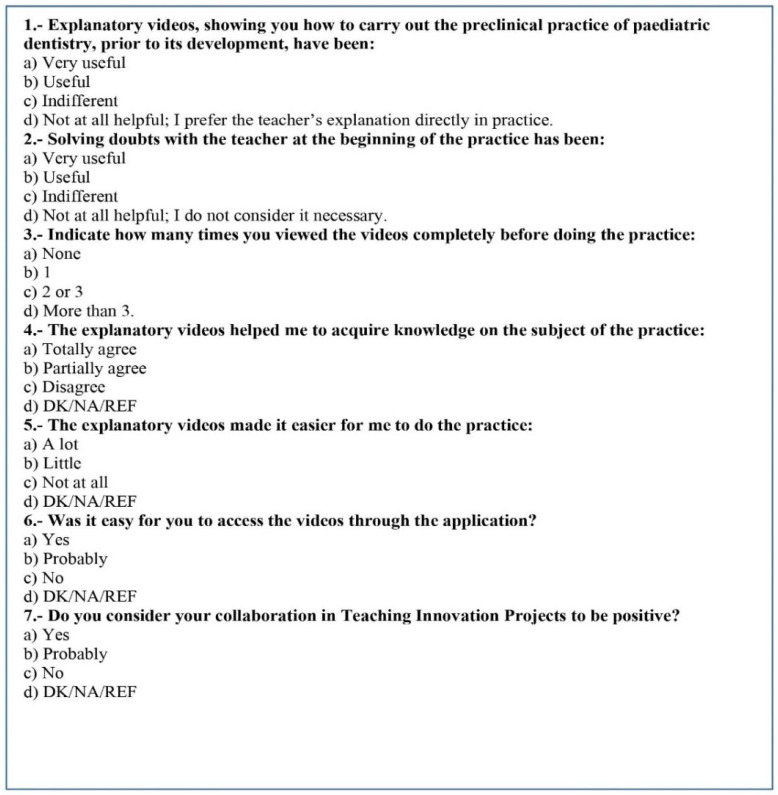
Satisfaction survey.

During the preclinical practice, the students resolved any doubts with the help of the teacher and performed the practice of pulpotomy in artificial temporary teeth. Once the practice was finished, the students were asked to give the teacher the teeth with the treatments carried out. To guarantee anonymity of the samples, each student was given a small bag identified with a number in which they entered their work. At the end of the practice, the students filled out the same knowledge questionnaire (Post-Q) as well as a satisfaction survey (Figure 1).

Four evaluation guides (0 to 1 points each) were established to carry out an objective evaluation (Table 2). A single teacher evaluated all the students, who was at no time aware of the group to which the samples belonged. The final grading range was 0–4. Once the first evaluation was done, another teacher repeated the grading procedure on 30 teeth. An Excel table was created in which the results of the evaluation were incorporated, and the statistical analysis was carried out using the SPSS program, version 25 (IBM Corp, Armonk, NY, USA).

The knowledge questionnaires were analysed concerning theoretical learning. To determine whether there were differences between groups A and B, the Mann–Whitney U-test was used for independent tests, comparing the increase in correct answers (average) in both groups. Fisher’s exact test was used to determine differences between the two groups in each of the questions. The McNemar test was also used to assess the learning of knowledge in the entire sample.

To assess practical learning, the ratings of pulpotomies performed on artificial teeth were taken into account. They were compared between the two groups, for which the non-parametric Kruskal–Wallis distribution test was used, and then a multiple comparison test was carried out “a posteriori” with Bonferroni correction. The Kappa test was applied to assess inter-examiner agreement and the Intraclass Correlation Coefficient (ICC) was applied to assess intra-examiner agreement.

The satisfaction survey was evaluated with Fisher’s exact test.

## 3. Results

Of the 50 students that formed the sample, the Post-Q response rate was 100% in group A and 92% in group B. The performance of pulpotomies was 100% in group A and 96% in group B. The main reason why some questionnaires were not recorded or teeth were not graded was that the students did not attend the preclinical practice.

Table 3 presents the means (Ms), standard deviations (SDs), as well as the maximum and minimum score of the three variables: tooth scores, Pre-Q responses, and Post-Q responses.

The analysis of differences in theoretical learning between groups A and B showed that, although in the Post-Q the number of correct answers had increased in both groups, there were no significant differences between groups in the average number of correct answers, and the score means for Pre-Q and Post-Q were very similar (Table 1 and Table 3). Additionally, when the differences were evaluated question by question, no significant differences were found (Table 1). The average mark of all Pre-Q students was 3.95, and rose to 7.45 for Post-Q. There was an increase in the number of correct answers in the Post-Q, which was significant in both groups (*p* < 0.001) (Table 1 and Table 3). When analysing the learning in each of the questions, the results were significant for eight of the 10 questions (nos. 1–3 and 6–10). However, in question nos. 4 and 5 there was no learning (Table 1), possibly due to the good results obtained in these questions in the Pre-Q (especially in no. 4 with 80.4% correct answers).

**Figure 2:**
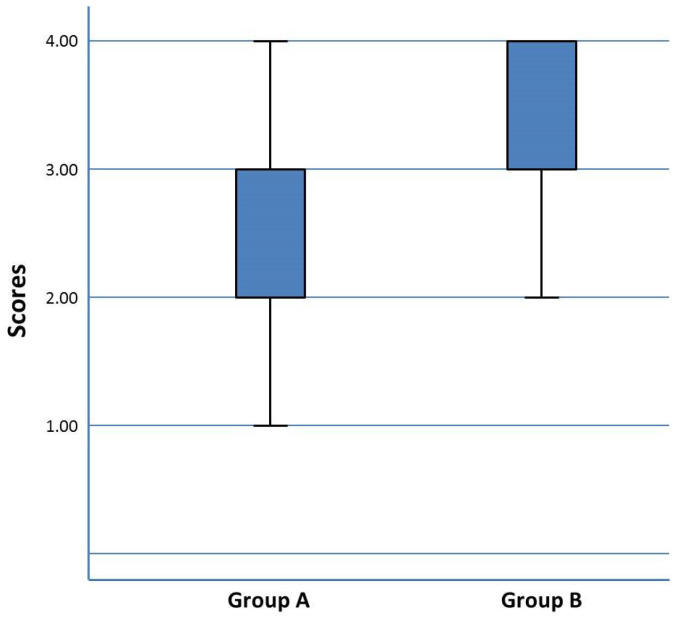
Qualifications of the practice of pulpotomy performed on artificial teeth (significant difference between groups; non-parametric Kruskal–Wallis distribution test, *p* < 0.001).

For tooth scores, inter-examiner agreement on all four rubrics was good or very good (0.7 for roof, 0.61 for walls, 0.88 for pulp tissue, and 0.82 for depth). The ICC was almost perfect (0.86) according to the scale proposed by Landis and Koch (Reference Landis and Koch). The grades for pulpotomies were higher in group B, which used Edpuzzle, compared to group A, which used Moodle (*p* = 0.001) (Figure 2).

In the satisfaction survey, there were significant differences only in the question of whether it had been easy and simple to access the videos, for which 100% of the students in group A answered that it had been very easy and simple, compared to 69.6% in group B that used Edpuzzle (*p* = 0.003). In group A, 92% found it very useful to have explanatory videos before the practices, while 78.2% of group B found the explanatory videos to be useful before the practices.

## 4. Discussion

The transfer of too much information in a short period of time causes cognitive overload. FL can contribute to a decrease in the level of cognitive load because it allows the student to gain knowledge about the topic before the lesson [6].

Hew et al., in a meta-analysis of 28 studies on the flipped classroom approach in health professions education, found a significant improvement in student learning compared to traditional teaching methods [7]. However, studies carried out on nursing and biology students found little difference in grades for the FL modality and the traditional model [8].

In another systematic review carried out by Gianoni-Capenakas et al. on the effectiveness and perception of FL suggested that it is a good methodology for transmitting knowledge to the student and stated that the greatest advantage is that the student assimilates information at their own pace [9].

In our previous work, we implemented FL in paediatric dentistry practices using videos through the Moodle platform with very satisfactory results. In response to the indications of the students, in this research, we wanted to incorporate another digital tool (i.e., Edpuzzle) through which they could view the videos and allow feedback between student and teacher on the selected platform. In addition, in this study we analysed both theoretical and practical learning, since this is the main objective of these preclinical classes [5].

Analysing our results in a general way showed that learning was improved by applying FL, regardless of the platform used—Moodle or Edpuzzle. These results coincide with those of other authors who applied FL in different branches of dentistry, verifying that this teaching method improves learning in anatomy, orthodontics, prosthetics and paediatric dentistry [5,10,11,12,13,14,15,16].

The use of instructional videos as teaching material to apply FL provided favourable results in this and other studies. It not only improves learning and reinforces students’ attitudes, but also increases their confidence when performing certain clinical techniques such as child-patient anaesthesia [16,17] and tooth preparation for crown placement [12,16,17].

Our results also indicated that the use of videos can help improve technical skills. However, Chen et al. found no difference between the groups that watched videos and the group that attended the lecture class [11]. In a systematic review on FL in dentistry, Vanka et al. verified that it improves student satisfaction, but cannot be guaranteed to improve grades or develop skills, so they suggest that it should be studied in more depth [18].

In our study, the grades obtained in the pulpotomies were higher in the Edpuzzle group than the Moodle group. Therefore, we believe that the choice of platform can influence learning. The major limitation of our study is the sample size. It would be interesting to enlarge the sample, but we are conditioned by the low number of students enrolled in our degree. Based on these preliminary results, we will continue studying to apply it in future courses.

Edpuzzle is designed for video teaching and provides useful tools not provided by Moodle. It allows teachers to manage videos at their convenience such as pausing, entering comments (in text or voice) and questions, which possibly focuses the student’s attention on its content. In addition, the display cannot be advanced, so that the student must see each lesson from beginning to end. The teacher can even choose to prevent the progress of the video content if the questions posed are not answered correctly. This motivates the student to revisualise the previous parts not assimilated or not understood.

On both platforms, Moodle and Edpuzzle, the teacher has individualised information per student about the number of views of the video. However, with Edpuzzle it is even possible to know which parts of the video have been viewed the most, allowing the teacher to locate the information that is most complex for the student so that it can be reinforced or expanded in the subsequent face-to-face class.

In addition, by allowing the addition of questions, and if programmed, Edpuzzle automatically evaluates the student, calculating the correct answers per class (video) and per course. This option is also not available in Moodle.

Evaluating the difference in learning between the group that used Moodle (A) and the one that used Edpuzzle (B) showed that, although the number of correct answers had increased in both groups in the Post-Q, there were significant differences between them in the increase in correct answers (average), since the average scores of both the Pre-Q and Post-Q were very similar in both groups. However, as previously mentioned, the ratings were higher in the Edpuzzle group. Karaka used Edpuzzle as a platform to apply FL and observed that when FL is well structured, it emerges as a way to reduce cognitive load, but we cannot directly compare our results with those of this or other authors because no literature compares these two platforms [19].

In group A, 92% found it very useful to have explanatory videos before the practices compared to 78.2% in group B. Therefore, we believe that our results are satisfactory, and consistent with those of other authors who used FL in different fields of dentistry [5,13,16,17,20,21,22]. We found significant differences between the two groups in terms of access to the videos, since 30.5% of the students in group B did not find it easy to access Edpuzzle. We believe that this could be justified because the students in group A had access through the CV, a tool with which they are very familiar and with which they usually work in the subject. However, accessing the video through the Edpuzzle link was somewhat unusual, although it was sent to the student’s institutional email in a timely manner.

## 5. Conclusions

Our results indicate that the use of videos to carry out FL improves learning, regardless of the platform used to apply it. However, the grades obtained in the practice of pulpotomies in artificial teeth were higher for students who used the Edpuzzle platform.

The tools provided by Edpuzzle for viewing the videos stimulate the attention and interest of the student, which implies improved learning. For the teacher, Edpuzzle makes it easy to create and customise videos, making it a very useful platform for using FL.

## Figures and Tables

**Table 1:** Correct answers to the pulpotomy knowledge questionnaire. The correct answer is shown in bold. Significant differences between Pre-Q and Post-Q (learning of each question: McNemar’s test: 1: *p* < 0.001; 2: *p* = 0.004).

QUESTIONNAIRE	GROUP A		GROUP B		TOTAL	
	PRE-Q	POST-Q	PRE-Q	POST-Q	PRE-Q	POST-Q
**1.** Pulpotomy in temporary dentition consists of: a) Partial removal of the coronal pulp. b) **Complete removal of the coronal portion of the dental pulp**. c) Complete removal of the coronal pulp and partial removal of the root pulp. d) Complete removal of the coronal and root pulp.	4	24	9	21	13	45 ^1^
**2.** The main objective of pulpotomy in temporary teeth is to: a) Completely eliminated caries. b) **Cure the tooth and maintain its vitality.** c) Treat the tooth, eliminating its vitality. d) Reconstruct the affected tooth and maintain it until exfoliation.	9	18	11	19	20	37 ^1^
**3.** When is it recommended to do a pulpotomy in temporary dentition? a) When the infection or pulp inflammation affects the entire pulp. b) Teeth with abscess or fistula but without symptoms. c) **When the infection or inflammation is limited to the coronal pulp.** d) All the previous answers are correct.	12	22	12	20	24	42 ^1^
**4.** Sequence to perform a pulpotomy in temporary dentition. Point out the correct answer: a) **Anaesthesia, absolute isolation, caries elimination, cameral opening, and removal of the pulp tissue.** b) Anaesthesia, elimination of caries, absolute isolation, cameral opening, and removal of the pulp tissue. c) Anaesthesia, caries removal, cameral opening, absolute isolation, and removal of the pulp tissue. d) Anaesthesia, isolation, cameral opening, removal of pulp tissue, and elimination of caries.	18	19	19	19	37	38
**5.** The removal of the pulp tissue will be carried out with (indicate the wrong one): a) Round bur at low speed. b) **Round bur at high speed.** c) With an excavator. d) a and c are correct.	8	8	4	7	12	15
**6.** The roots of the temporary teeth, in relation to the size of the crown are: a) Longer and thicker than those of permanent teeth. b) Shorter and thicker than those of permanent teeth. c) **Longer and thinner than those of permanent teeth**. d) Shorter and thinner than those of permanent teeth.	5	11	5	16	10	27 ^1^
**7.** We will control the bleeding from the root pulp with: a) Absorbent haemostatic sponges. b) Washing with plenty of water and then drying with air. c) **Pressing with a cotton ball.**. d) Waiting a few minutes for the bleeding to stop.	19	24	12	21	31	45 ^2^
**8.** Once the pulpotomy is done, in this preclinical practice, what material do we use to fill the cavity? a) Glass ionomer cement. b) Composite resin. c) Silver amalgam. d) **Zinc oxide eugenol cement.**	7	20	6	18	13	38 ^1^
**9.** Regarding the technique of performing a pulpotomy, indicate which answer is false: a) Caries will be eliminated before coronal opening. b) After performing the pulpotomy and filling the cavity, it is recommended to place a preformed crown. c) **The removal of the pulp will be done with a lance bur at low speed or with an excavator**. d) Once haemostasis of the root pulp has been achieved, we will observe the entry into the root canals.	5	20	7	17	12	37 ^1^
**10.** The occlusal fossa of the primary molars has a thickness of dentin: a) **Greater than the permanent molars.** b) Less than the permanent molars. c) The same as the permanent molars d) There is no dentin in the occlusal fossa.	8	19	4	15	12	34 ^1^

**Table 2:** Evaluation guide.

INDICATORS	Entire Roof of the Pulp Chamber Has Been Removed	Cavity Walls Are Too Thin	All Chamber Pulp Tissue Has Been Removed	Depth of the Cavity Exceeds the Pulp Chamber
**INCORRECT** **(0 points)**	No	Yes	No	Yes
**CORRECT** **(1 point)**	Yes	No	Yes	No

**Table 3:** Descriptive statistics of the study (M, mean; SD, standard deviation).

	GROUP A					GROUP B				
	N		M ± SD			N		M ± SD		
	Valid	Lost		Min	Max	Valid	Lost		Min	Max
**EXAMINED TEETH**	25	0	2.64 ± 0.95	1	4	24	1	3.55 ± 0.59	2	4
**PRE-Q**	23	2	4.13 ± 1.82	1	7	23	2	3.87 ± 0.82	1	6
**POST-Q**	25	0	7.40 ± 1.53	4	10	23	2	7.52 ± 1.82	5	10

## Data Availability

The data presented in this study are available on request from the corresponding author.

## References

[1] Bergmann J., Sams A. (2012). Flip Your Classroom: Reach Every Student in Every Class Every Day.

[2] Singh V., Abdellahi S., Maher M.L., Latulipe C. (2016). The video collaboratory as a learning environment. Proceedings of the 47th ACM Technical Symposium on Computer Science Education.

[3] Baker A. (2016). Active Learning with Interactive videos: Creating student-guided learning materials. J. Libr. Inf. Serv. Distance Learn..

[4] Ros I. (2008). Moodle, la Plataforma para la Enseñanza y Organización Escolar. Ikastorratza, e-Revista de Didáctica 2. http://www.ehu.es/ikastorratza/2_alea/moodle.pdf.

[5] Gallardo N.E., Caleya A.M., Sánchez M.E., Feijóo G. (2022). Learning of paediatric dentistry with the flipped classroom model. Eur. J. Dent. Educ..

[6] Abeysekera L., Dawson P. (2015). Motivation and cognitive load in the flipped classroom: Definition, rationale and a call for research. High. Educ. Res. Dev..

[7] Hew K.F., Lo C.K. (2018). Flipped classroom improves student learning in health professions education: A meta-analysis. BMC Med. Educ..

[8] Sabater-Mateu M.P., Curto-García J.J., Rourera-Roca À., Ferrer M.C.O., Abós S.C., Ibáñez S.C., del Pino Gutiérrez A. (2017). Aula invertida: Experiencia en el Grado de Enfermería. Revista d’Innovació Docent Universitària.

[9] Gianoni-Capenakas S., Lagravere M., Pacheco-Pereira C., Yacyshyn J. (2019). Effectiveness and Perceptions of Flipped Learning Model in Dental Education: A Systematic Review. J. Dent. Educ..

[10] Chutinan S., Riedy C.A., Park S.E. (2018). Student performance in a flipped classroom dental anatomy course. Eur. J. Dent. Educ..

[11] Chen M.S., Horrocks E.N., Evans R.D. (1998). Video versus lecture: Effective alternatives for orthodontic auxiliary training. Br. J. Orthod..

[12] Robinson P.B., Lee J.W. (2001). The use of real time video magnification for the pre-clinical teaching of crown preparations. Br. Dent. J..

[13] Varthis S., Anderson O.R. (2018). Students’ perceptions of a blended learning experience in dental education. Eur. J. Dent. Educ..

[14] Bohaty B.S., Redford G.J., Gadbury-Amyot C.C. (2016). Flipping the classroom: Assessment of strategies to promote student-centered, self-directed learning in a dental school course in pediatric dentistry. J. Dent. Educ..

[15] Gadbury-Amyot C.C., Redford G.J., Bohaty B.S. (2017). Dental students’ study habits in flipped/blended classrooms and their association with active learning practice. J. Dent. Educ..

[16] Kenny K.P., Alkazme A.M., Day P.F. (2018). The effect of viewing video clips of paediatric local anaesthetic administration on the confidence of undergraduate dental student. Eur. J. Dent. Educ..

[17] Wong G., Apthorpe H.C., Ruiz K., Nanayakkara S. (2019). An innovative educational approach in using instructional videos to teach dental local anaesthetic skilss. Eur. J. Dent. Educ..

[18] Vanka A., Vanka S., Wali O. (2020). Flipped classroom in dental education: A scoping review. Eur. J. Dent. Educ..

[19] Karaca C., Ocak M.A. (2017). Effect of Flipped learning on cognitive load: A higher education research. J. Learn. Teach. Digit. Age.

[20] Park S.E., Howell T.H. (2015). Implemention of a FC educational model in a predoctoral dental course. J. Dent. Educ..

[21] Shapiro M.C., Anderson O.R., Lal S. (2014). Assessment of a novel module for training dental students in child abuse recognition and reporting. J. Dent. Educ..

[22] Faraone K.L., Garret P.H., Romberg E. (2013). A blended learning approach to teaching pre-clinical complete denture prosthodontics. Eur. J. Dent. Educ..

